# Direct regulation of the cardiac ryanodine receptor (RyR2) by O-GlcNAcylation

**DOI:** 10.1186/s12933-023-02010-3

**Published:** 2023-10-13

**Authors:** Chidinma A Okolo, Ei-Phyo Khaing, Valeria Mereacre, Rachel S Wallace, Michelle L Munro, Jeffrey R Erickson, Peter P. Jones

**Affiliations:** 1 Department of Physiology, School of Biomedical Sciences, Division of Health Sciences, and HeartOtago, University of Otago, Dunedin, Otago, New Zealand; 2 Beamline B24, Life Sciences Division, Diamond Light Source, Harwell Science and Innovation Campus, Didcot, OX11 0DE England, United Kingdom

**Keywords:** Ryanodine receptor (RyR2), O-GlcNAcylation, SOICR, Diabetes

## Abstract

**Background:**

O-GlcNAcylation is the enzymatic addition of a sugar, O-linked β-N-Acetylglucosamine, to the serine and threonine residues of proteins, and is abundant in diabetic conditions. We have previously shown that O-GlcNAcylation can trigger arrhythmias by indirectly increasing pathological Ca^2+^ leak through the cardiac ryanodine receptor (RyR2) via Ca^2+^/calmodulin-dependent kinase II (CaMKII). However, RyR2 is well known to be directly regulated by other forms of serine and threonine modification, therefore, this study aimed to determine whether RyR2 is directly modified by O-GlcNAcylation and if this also alters the function of RyR2 and Ca^2+^ leak.

**Methods:**

O-GlcNAcylation of RyR2 in diabetic human and animal hearts was determined using western blotting. O-GlcNAcylation of RyR2 was pharmacologically controlled and the propensity for Ca^2+^ leak was determined using single cell imaging. The site of O-GlcNAcylation within RyR2 was determined using site-directed mutagenesis of RyR2.

**Results:**

We found that RyR2 is modified by O-GlcNAcylation in human, animal and HEK293 cell models. Under hyperglycaemic conditions O-GlcNAcylation was associated with an increase in Ca^2+^ leak through RyR2 which persisted after CaMKII inhibition. Conversion of serine-2808 to alanine prevented an O-GlcNAcylation induced increase in Ca^2+^ leak.

**Conclusions:**

These data suggest that the function of RyR2 can be directly regulated by O-GlcNAcylation and requires the presence of serine-2808.

**Supplementary Information:**

The online version contains supplementary material available at 10.1186/s12933-023-02010-3.

## Background

The cardiac ryanodine receptor (RyR2) is responsible for the release of Ca^2+^ from the sarcoplasmic reticulum (SR) to drive cardiac contraction. This mechanism is commonly known as Ca^2+^ -induced Ca^2+^ release (CICR) as Ca^2+^ is released from RyR2 in response to Ca^2+^ influx through L-type Ca^2+^ channels [1]. In addition to this physiological process, RyR2 can also be activated by Ca^2+^ within the SR in a process termed store overload induced Ca^2+^ release (SOICR) [2]. SOICR is strongly associated with cardiac arrhythmias and heart failure [3].

RyR2 can be regulated by post-translational modifications such as phosphorylation and oxidation [4–7]. Of these, phosphorylation is perhaps the most well-studied, with three sites identified: S2030, S2808 and S2814 [8]. These sites are targeted by protein kinase A (PKA), Ca^2+^/calmodulin dependent kinase II (CaMKII) and protein kinase G (PKG). Phosphorylation of these sites enhances Ca^2+^ release during CICR, and in excess, promotes SOICR [4, 5].

An emergent post-translational modification associated with regulation of cardiac function and Ca^2+^ cycling is O-GlcNAcylation (O-GlcNAc) [9]. O-GlcNAcylation is the enzymatic addition of a sugar, O-linked β-N-Acetylglucosamine, to the serine and threonine residues of proteins, which are also modified by phosphorylation [10]. Hence it has been suggested that there is an interlink between O-GlcNAcylation and phosphorylation [9, 10]. O-GlcNAcylation is regulated by two highly conserved enzymes, O-GlcNAc transferase (OGT) and O-GlcNAcase (OGA) which add and remove the modification, respectively [9]. The precursor of O-GlcNAcylation, uridine diphosphate N-acetylglucosamine (UDP-GlcNAc), is derived from the glucose metabolism via the hexosamine biosynthetic pathways [11]. Therefore, it is unsurprising that O-GlcNAcylation is abundant in diabetic conditions [11, 12].

We have previously shown that O-GlcNAcylation can increase SOICR indirectly through the activation of CaMKII and subsequent phosphorylation of RyR2 [12]. Indeed, the increased O-GlcNAcylation of CaMKII and subsequent phosphorylation of RyR2 is already established to drive arrhythmias in diabetes, suggesting that prevention of CaMKII O-GlcNAcylation may be a therapeutic strategy [13]. However, although previous studies have clearly shown a role of O-GlcNAcylation of CaMKII in increasing SOICR, the inhibition of CaMKII under these conditions does not completely ameliorate the effect [12]. Therefore, other mechanisms must exist. RyR2 is a very large protein with multiple surface exposed serine and threonine residues capable of accepting O-GlcNAcylation modification, including the phosphorylation sites described above. To the best of our knowledge there are no studies examining the O-GlcNAcylation of cardiac RyR2 in diabetes. To date, O-GlcNAcylation of RyR2 has only been identified in the brain and linked to Alzheimer’s disease [14]. Moreover, there have been no functional studies examining direct O-GlcNAcylation of RyR2 in any cell or tissue type.

In this study we used a human embryonic kidney (HEK) 293 cell model, stably expressing RyR2, to determine the CaMKII independent effect of O-GlcNAcylation on SOICR. Next, we used site directed mutagenesis to establish the sites within RyR2 to mediate this effect. Our data show that RyR2 can be dynamically modified by O-GlcNAcylation and that this is associated with an increase in SOICR, independent of CaMKII activity. These findings suggest that O-GlcNAcylation of RyR2 promotes SOICR and is likely to contribute to the occurrence of arrhythmias seen in the diabetic heart. As O-GlcNAcylation of RyR2 has also been observed in the brain it is also likely to have a role there too.

## Methods

### Human right atrial appendages (RAA)

Human RAA were obtained from consenting patients (ethics: LRS/12/01/001/AM17), with or without Type 2 Diabetic Mellitus (T2DM), undergoing on-pump coronary artery bypass graft surgery. Patient characteristics are shown in Table 1. Patients with pre-operative arrhythmia were excluded from the study.

**Table 1:** **Patient characteristics** Patients were matched for age, ejection fraction and body mass index (BMI). Mean ± SEM shown, n = 8 per group, statistical analysis was performed using a Welch’s t test

Parameter	Non-diabetic	Diabetic	P value
Age (years)	61.40 ± 2.4	69.11 ± 3.4	0.12
Ejection Fraction (%)	57.49 ± 1.4	58.78 ± 3.02	0.81
BMI (kg/m2)	31.27 ± 1.53	29.71 ± 2.04	0.43
HbA1c (mmol/L)	36.5 ± 0.82	56.9 ± 3.01	< 0.0001

### Zucker diabetic fatty (ZDF) rats

Left ventricular tissue samples were collected from 5-month-old male ZDF rats or their lean littermate controls. Rats were housed at 20 ± 1 °C under a 12 h light–dark cycle and provided with food and water ad libitum. Animals were maintained on a Purina 5008 diet (LabDiet, St. Louis, MO, USA). Diabetic status was confirmed using a glucometer (Roche, Basel, Switzerland) when the hearts were removed. All procedures and animal handling were conducted with the approval of the University of Otago Animal Ethics Committee (AUP 19–250) in accordance with the New Zealand Animal Welfare Act (1999).

### Maintenance of stable inducible HEK 293 cell lines

Stable inducible human embryonic kidney (HEK 293) cells expressing wild-type RyR2 and S2808A were generated as previously described [5]. Cells were grown in grown in low (5.5 mM) or high glucose (25 mM) supplemented Dulbecco’s Modified Eagle Medium (DMEM) (Life technologies). Where required, RyR2 expression was induced through the application of 0.1 µg/mL tetracycline 16–18 h before experiment. In cells without RyR2 expression tetracycline was omitted from this step. To either promote or inhibit O-GlcNAcylation, 100 nM of Thiamet-G (Thm-G) or 50 µM of Diazo-6-oxornleucine (DON), respectively [12], were added to the media at the time of induction of RyR2 expression when required.

### Preparation of protein lysates

Protein lysates were prepared from HEK293 cells as described previously [5, 15]. In brief, cells were collected in a phosphate-buffered saline (PBS) containing 0.5 mM EDTA by centrifugation at 3000 g for 10 min. The cells were then resuspended in a cell lysis buffer containing 137 mM NaCl, 25 mM Tris/HEPES (pH 7.4), 1% CHAPS, 0.5% soybean phosphatidylcholine, 1 mM benzamide, and protease inhibitors which includes 2 µg/ml leupeptin, 2 µg/mL aprotinin, 2 µg/mL pepstatin A, 2.5 mM dithiothreitol and 0.5 mM phenylmethylsulfonyl fluoride. The cell lysates were incubated on ice for 1 h before 2x centrifugation at 16,000 g for 30 min at 4 °C. The resulting supernatants were stored at -80 °C until required.

To prepare protein lysates from human RAA and ZDF rats, approximately 100 mg of tissue was added to 100 µL of stainless-steel beads (Lab supply) plus 300 µL of radioimmuno precipitation assay (RIPA) buffer containing 50 mM Tris-HCl (pH 7.4), 150 mM NaCl, 0.1% SDS, 1% Triton X-100, 1 mM EDTA and protease inhibitors (as described above). The tissues were homogenized using a Bullet blender® (Next Advance) at speed 8 for 4 min followed by centrifugation at 15,000 g for 30 min at 4 °C. The supernatant was stored at -80 °C until required [16, 17].

### Immunoprecipitation

Briefly, 40 µg of protein lysates, prepared as described above, were incubated with protein G-Sepharose beads (15 µl) that were pre-bound with 1 µl total-RyR2 antibody (34 C) (Abcam) at 4 °C for 18–20 h. The supernatant was removed and retained, and the beads were washed 3x in PBS before storage at -80 °C until required.

### Sodium dodecyl polyacrylamide gel electrophoresis (SDS-PAGE) and western blotting

Briefly, 40 µg of protein lysates, or immunoprecipitated samples (beads and supernatant), were solubilized in Laemmli sample buffer before separation via 6% (RyR2) or 12% (GAPDH) SDS-PAGE. Following separation, proteins were transferred onto a 0.45 μm nitrocellulose membrane. The membranes were then blocked with PBS containing 0.5% Tween 20 (PBS-T) and 5% (w/v) dried non-fat skimmed milk powder for 1 h before protein levels were then detected with specific antibodies for total-RyR2 (34 C) (1:1000) (Abcam), total O-GlcNAc, clone 9D1.E4(10) (1:1000) (Abacus) which recognizes all O-GlcNAc modified proteins, and GAPDH (1:3000) (GeneTex). This was followed by detection with the appropriate peroxidase-coupled secondary antibody (1:10000) (Abcam). All bands were visualised using SuperSignal West Pico Plus Chemiluminescent Substrate (ThermoFisher Scientific) via a chemiDoc MP Imaging system (Bio-rad). The band intensities were analysed using ImageJ. O-GlcNAc modified RyR2 was normalised to total RyR2 levels.

### Cytosolic Ca^2+^ imaging and analysis

Imaging was performed as described previously [2]. To permit Ca^2+^ measurements, the cells were loaded with 2 µM Fluo-4 before imaging. Cells were then continuously superfused with Krebs-Ringers HEPES (KRH) solution containing: 125 mM NaCl, 5 KCl, 25 mM HEPES, 5.5 mM glucose, 1.2 mM MgCl_2_ and variable CaCl_2_ (0–1 mM) (pH 7.4) at room temperature. When required, the drugs of interest were applied throughout the superfusion of the cells (100 nM Thm-G (O-GlcNAc promotor), 50 µM DON (O-GlcNAc inhibitor) and/or 2 µM of KN93 or KN92 (CaMKII inhibitor and inactive analogue). At the end of the experiments, 20 mM caffeine was applied to deplete intracellular Ca^2 +^ store (the endoplasmic reticulum in HEK293 cells). Fluo-4 AM dye was excited at 470 nm (40 nm bandwidth) every 2 s with an exposure time of 100 ms using a pE-4000 CoolLED system (Coherent Scientific Pty. Ltd, Australia). Fluorescence of Fluo-4 was detected using a Zyla 4.2 PLUS sCMOS camera (Andor) through a long pass dichroic mirror (495 nm) and emission filter (> 515 nm). Data capture and analysis were performed using NIS-Elements AR 4.00.03 64-bit software (Coherent Scientific Pty. Ltd, Australia). The changes of Fluo-4 fluorescence expressed as F/F_0_, where F denotes the fluorescence intensity at a given time, while F_0_ is the average fluorescence intensity recorded in the first 30 s.

### Luminal Ca^2+^ imaging and analysis

Luminal Ca^2+^ was measured in cells grown as above with the additional transfection of 2 µg of the calcium-measuring organelle-entrapped protein indicator (CEPIA) cDNA [18]. CEPIA transfection was performed, using Ca^2+^ phosphate precipitation, 24 h before RyR2 induction. During imaging the cells were perfused continuously at room temperature with KRH containing various concentrations of Ca^2+^ (0 and 2 mM), tetracaine (2 mM to block RyR2 from releasing Ca^2+^) and caffeine (20 mM to deplete the SR store) [5]. O-GlcNAcylation and CaMKII activity were manipulated as described above. Excitation was by a pE-4000, CoolLED at 580 nm (20 nm bandwidth) at 2 s intervals. Fluorescent signals were recorded with an Zyla 4.2 PLUS sCMOS camera (Andor) at 640 nm emission wavelength with a 50 ms exposure. Data capture and analysis were performed using NIS-Elements AR 4.00.03 64-bit software (Coherent Scientific Pty. Ltd, Australia). Store size = F_max_ – F_min_, Release threshold = (F_SOICR_ – F_min_) / Store size x 100%, Termination threshold = (F_termi_ – F_min_) / Store size x 100% and Fractional release = Release – Termination threshold.

### Statistical analysis

Results are presented as mean ± SEM. Statistical analysis is described within the corresponding figure legends. Differences were considered statistically significant if p < 0.05. All plotting, data analysis and curve fittings were performed using GraphPad Prism 10.0.2 (GraphPad, La Jolla, CA).

## Results

### RyR2 is O-GlcNAcylated

**Fig. 1:**
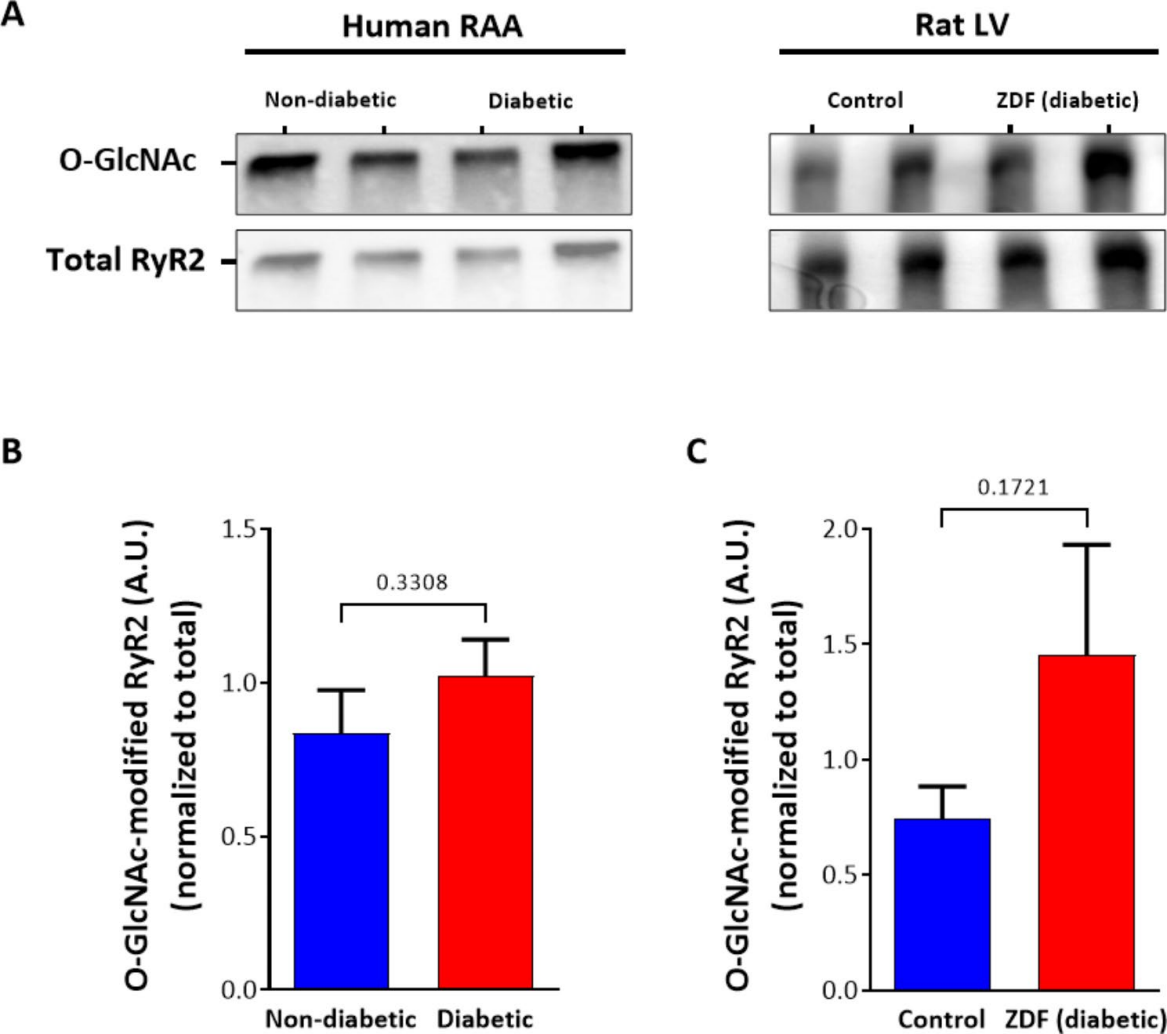
RyR2 is O-GlcNAcylated. (**A**) Representative blots showing total O-GlcNAc-RyR2 and total RyR2 expression from diabetic and non-diabetic human right atrial appendages (RAA) and left ventricles of Zucker diabetic fatty (ZDF) rat and non-diabetic lean (control) rats. The samples are labelled above the corresponding lane. O-GlcNAc modified RyR2 normalised to total RyR2 expression in (**B**) human RAA samples (n = 8 per group) and, (**C**) rat LV samples (n = 9 per group). Data displayed as Mean ± SEM. Statistical analysis was performed using a Welch’s t test

We have previously reported that O-GlcNAcylation can modify the activity of RyR2 indirectly via an increase in the activity of CaMKII and subsequent phosphorylation of RyR2 [12]. However, whether RyR2 is also directly regulated by O-GlcNAcylation in the heart is unknown. Using O-GlcNAc-specific antibody, we identified, for the first time, that O-GlcNAcylation of RyR2 occurs in both human RAA and rat left ventricular tissue (Fig. 1A). Neither species showed a significant increase in O-GlcNAcylation in the diabetic group. However, a trend toward an increase was evident in the diabetic rats (p = 0.17, Fig. 1B). To confirm the O-GlcNAc band corresponding to the Mw of RyR2 was indeed O-GlcNAcylated RyR2, we immunoprecipitated RyR2 from human RAA tissue. As shown in Supplementary Figure S1, immunoprecipitated RyR2 was detected by the O-GlcNAc antibody, whereas the supernatant from which RyR2 was immunoprecipitated no longer contained an O-GlcNAc identified band at the same Mw. Thus, we were able to confirm the identify the O-GlcNAcylation of RyR2 in the absence of a RyR2-O-GlcNAcylation specific antibody.

### O-GlcNAcylation of RyR2 increases the propensity for SOICR

**Fig. 2:**
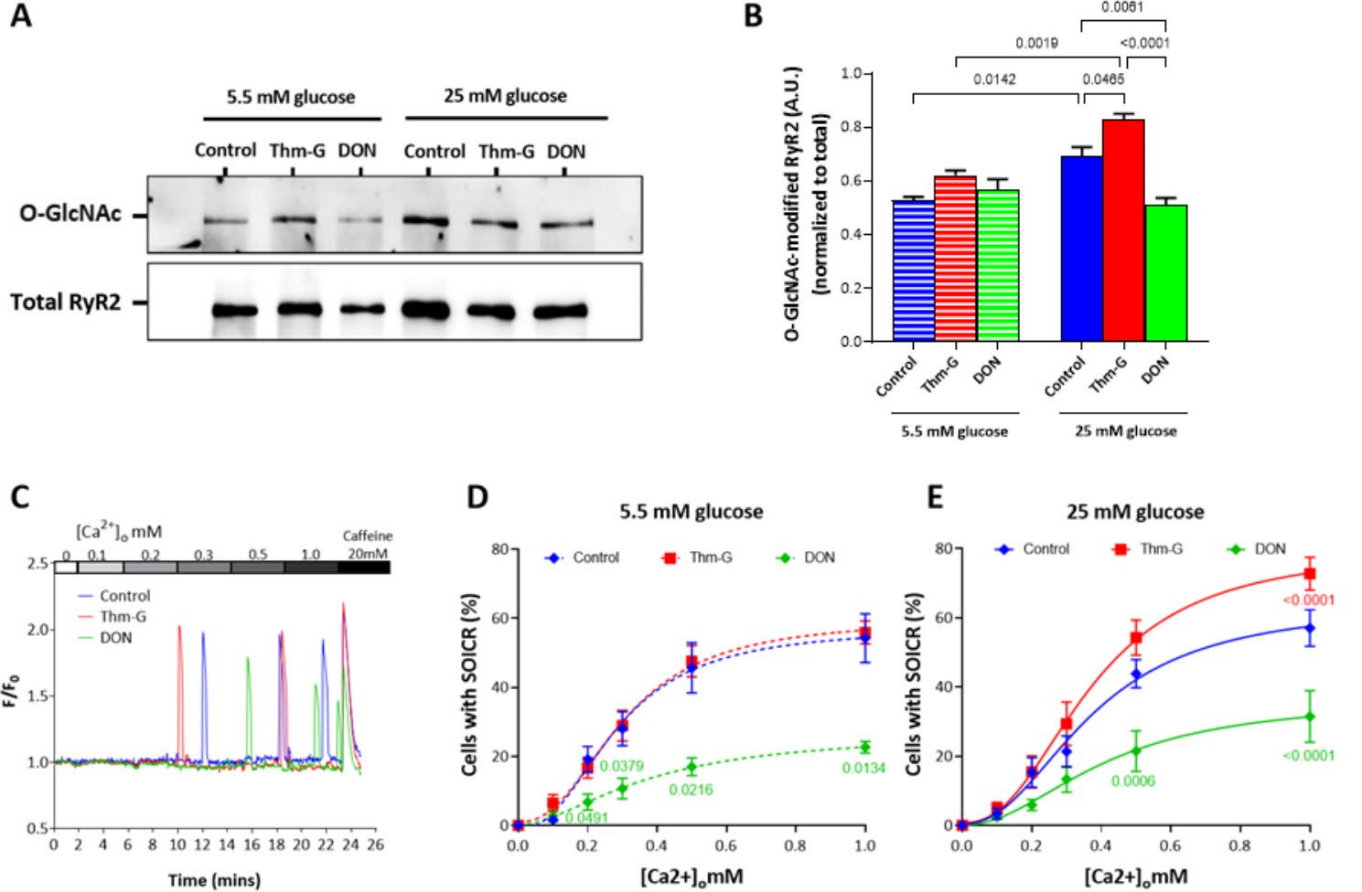
Changes in RyR2 O-GlcNAcylation are associated with changes in SOICR. Application of Thm-G and DON on the O-GlcNAc-RyR2 level in cells grown in low glucose (5.5 mM) and high glucose (25 mM) media. (**A**) Representative blots showing O-GlcNAc-RyR2 and total RyR2. (**B**) Densitometric analysis of O-GlcNAc-RyR2 level. All O-GlcNAc-RyR2 was normalised to total RyR2. Results are shown as Mean ± SEM. n = 3 independent lysates. Statistical analysis was performed using two-way ANOVA (Šidák post-hoc test). (**C**) Representative Fluo-4 traces presented as F/F_0_. Peaks represent SOICR events. (**D**) Percentage of cells experiencing SOICR at 5.5 mM glucose, (**E**) percentage of cells experiencing SOICR at 25 mM glucose. Data displayed are the average of 5–8 independent experiments and expressed as Mean ± SEM. Only significant P-values, vs. control are shown. Statistical analysis was performed using two-way ANOVA (Tukey post-hoc test)

Upon confirmation that RyR2 is directly O-GlcNAcylated, we next determined the impact of O-GlcNAcylation on the function of RyR2 using our well-established HEK 293 cell model. These cells have been extensively characterised, show spontaneous RyR2 activity comparable to cardiac cells and have served to investigate the impact of other post-translational modifications of RyR2 [5, 7]. To manipulate the level of O-GlcNAcylation, cells were grown in low (5.5 mM) or high (25 mM) glucose in the presence or absence of Thm-G or DON, a O-GlcNAcylation promotor and inhibitor, respectively. Figure 2 A confirms that RyR2 is O-GlcNAcylated in HEK 293 cells and that the level of O-GlcNAcylation can be manipulated. At 5.5 mM glucose, the addition of Thm-G or DON did not change the level of O-GlcNAc from baseline levels (Fig. 2B). However, in the presence of 25 mM glucose, the addition of Thm-G increased (p = 0.0456) and DON decreased (p = 0.0061) O-GlcNAcylation (Fig. 2B). The level of RyR2 O-GlcNAcylation was also higher in cells grown in 25 mM glucose compared to 5.5 mM glucose (p = 0.0019) (Fig. 2B). To further confirm the identity of the O-GlcNAc band corresponding to the Mw of RyR2 represents O-GlcNAcylated RyR2, we also performed the same experiment using HEK293 cells not induced to express RyR2. As shown in Supplementary Figure S2, when RyR2 expression was not induced in the HEK293 cell model no corresponding band was visible in the O-GlcNAc labelled samples.

To determine if these changes in RyR2 O-GlcNAcylation levels were associated with changes in SOICR, single cell Ca^2+^ imaging was performed (Fig. 2C). At 5.5 mM glucose, Thm-G had no effect on the occurrence of SOICR, whereas DON resulted in a significant reduction (Fig. 2D). At 25 mM glucose, both promotion and inhibition of O-GlcNAcylation altered the occurrence of SOICR. Thm-G significantly increased SOICR, whereas DON reduced its occurrence (Fig. 2E). These changes were consistent with the changes in O-GlcNAcylation of RyR2 seen in Fig. 2B.

### O-GlcNAcylation increases the propensity for SOICR in the absence of CaMKII

**Fig. 3:**
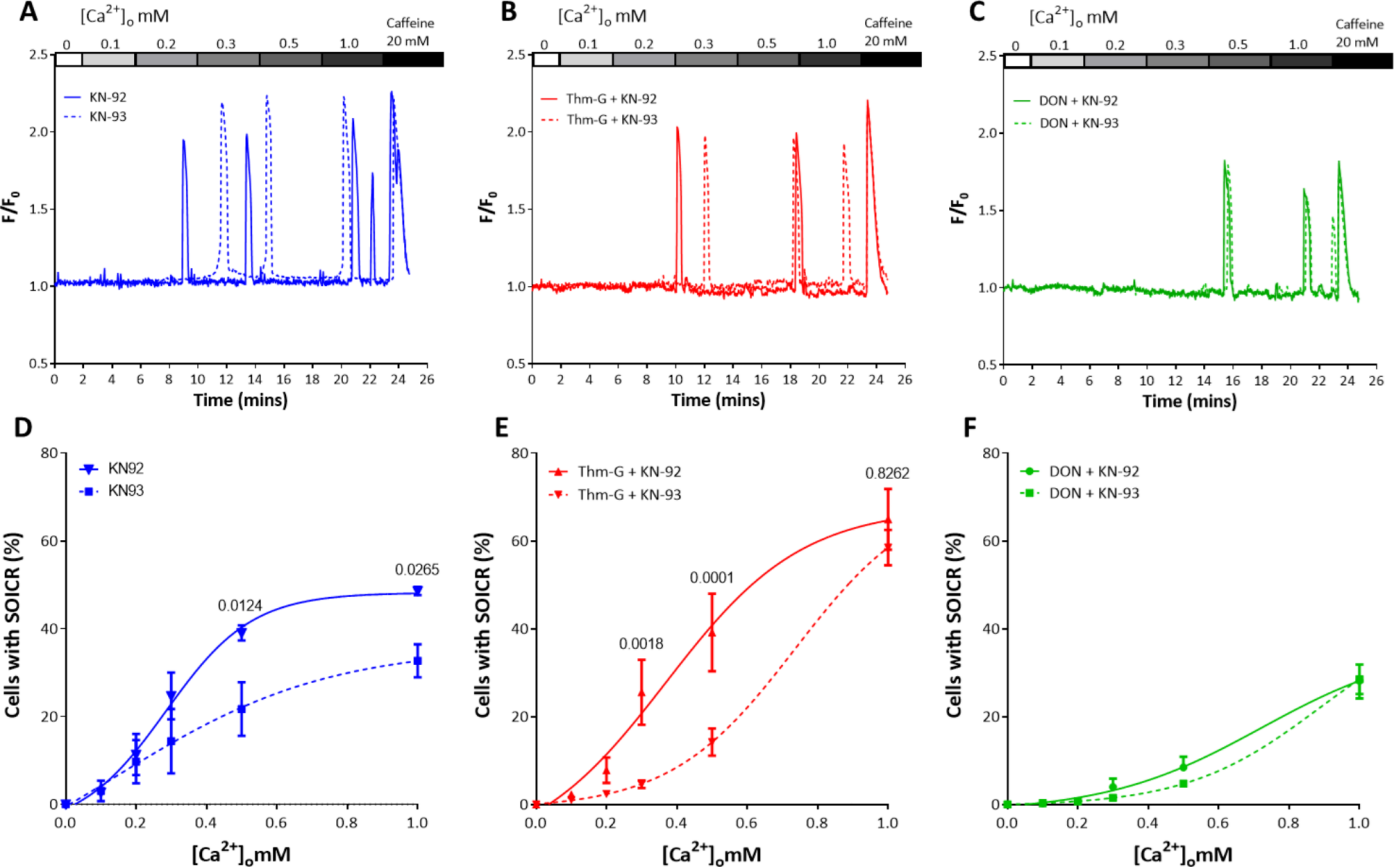
CaMKII inhibition does not prevent an O-GlcNAc mediated increase SOICR propensity. (**A-C**) Representative Fluo-4 traces (F/F_0_) of cells treated with KN-92 or KN-93 in the absence (**A**) or presence of Thm-G (**B**) or DON (**C**). Peaks represent SOICR events. (**D-F**) Percentage of cells experiencing SOICR treated with KN-92 or KN-93 in the absence (**D**) or presence of Thm-G (**E**) or DON (**F**). Data displayed are the average of 4–12 independent experiments and expressed as Mean ± SEM. P-values shown are for comparison of cells treated with KN-92 vs. KN-93. Statistical analysis was performed using two-way ANOVA (with Šidák post-hoc test)

As we have previously shown that O-GlcNAcylation can increase SOICR via the activation of CaMKII [12] and subsequent phosphorylation of RyR2, we next performed the same assay in the presence of the CaMKII inhibitor KN-93. As O-GlcNAcylation occurred and could be manipulated more easily at high glucose levels all following experiments were performed using 25 mM glucose. Under basal O-GlcNAc levels (not modified by Thm-G or DON) CaMKII inhibition reduces the occurrence of SOICR (Fig. 3A and D). Similarly, Fig. 3B and E show that when O-GlcNAcylation is promoted by the presence of Thm-G, inhibition of CaMKII still reduces occurrence of SOICR at lower Ca^2+^ concentrations (0.3–0.5 mM) compared to cells exposed to Thm-G + KN-92 (control) (p = 0.0018 and 0.0001, respectively). However, at 1mM Ca^2+^, the occurrence of SOICR was not affected by the presence of KN-93 (p = 0.8262) indicating that O-GlcNAcylation mediated changes in the occurrence of SOICR are largely CaMKII independent at this Ca^2+^ level. In the absence of O-GlcNAcylation (DON), the inhibition of CaMKII did not alter the occurrence of SOICR (Fig. 3 C and F).

### O-GlcNAcylation of RyR2 independent of CaMKII decreases the release threshold for SOICR

To determine how O-GlcNAcylation increased SOICR independently of CaMKII at higher Ca^2+^ concentrations, we next measured the threshold for SOICR using the intra-luminal Ca^2+^ indicator CEPIA. Our previous work has shown that a common mechanism by which the propensity of SOICR is increased is due to a reduction in the release threshold for SOICR (F_SOICR_) [5, 7]. We also examined the termination threshold, the intracellular Ca^2+^ store level (expressed as a percentage of the total store) at which SOICR events terminate (F_Termi_). Measuring F_SOICR_ and F_Termi_ also allows us to determine whether O-GlcNAcylation alters the magnitude of SOICR (fractional release) [15].

**Fig. 4:**
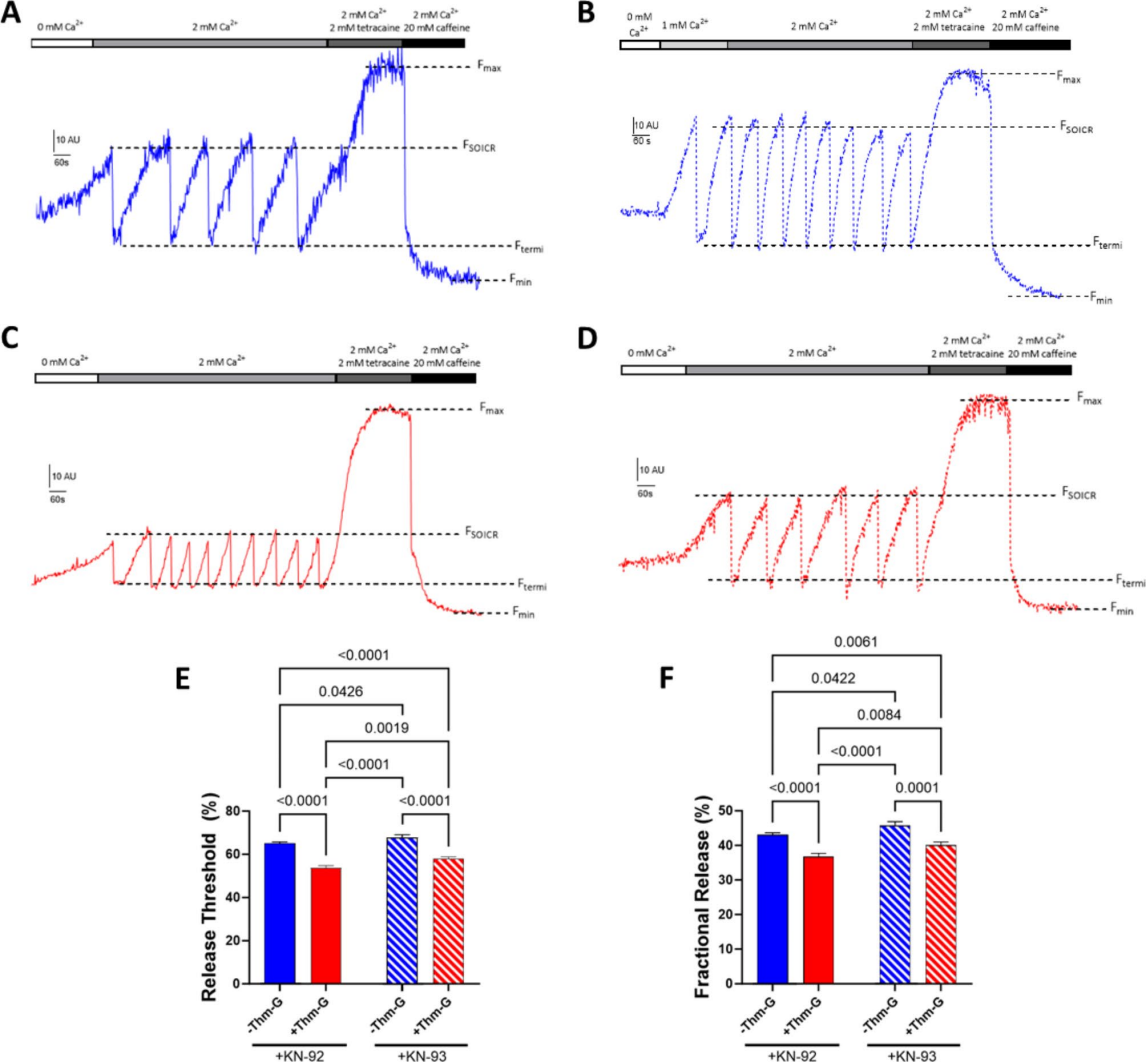
Effect of Thm-G and CaMKII inhibition on the characteristics of SOICR. Representative CEPIA traces for (**A**) KN-92 treated cells, cells treated with (**B**) KN-93, (**C**) Thm-G + KN-92 and (**D**) Thm-G + KN-93. Scale bar shows fluorescence (10AU) vs. time (60s). (**E**) Comparison of F_SOICR_, and (**F**) fractional release between treatments. Data were obtained from 156–542 cells recorded over 6–14 independent experiments. Results are expressed as Mean ± SEM. Statistical analysis was performed using a two-way ANOVA (with Fisher’s LSD post-hoc test)

Figure 4 A shows representative CEPIA traces from cells expressing RyR2 treated with 25mM glucose in the presence or absence of Thm-G, with (KN-93) or without (KN-92) CaMKII inhibition. In Fig. 4, F_max_ represents the maximum Ca^2+^ store in the presence of 2 mM tetracaine and F_min_ represents the minimum Ca^2+^ store in the presence of 20 mM caffeine. The free Ca^2+^ store size of each cell was obtained by F_max_-F_min_. F_SOICR_ was determined by the mean fluorescence immediately prior to each SOICR event (downward deflection), F_Termi_ was determined by the mean fluorescence of the nadir of SOICR. The release threshold (Fig. 4E) and fractional release (Fig. 4F) are reported as a percentage of free Ca^2+^ store as previously described [15]. As shown in Fig. 4 Thm-G (+ KN-92) significantly decreases the release threshold (F_SOICR_) and fractional release compared to RyR2 in the absence of Thm-G (both p < 0.0001). Importantly, when treated with the CaMKII inhibitor KN-93, Thm-G retains a significant effect on SOICR (p < 0.0001 and 0.001 for F_SOICR_ and fractional release, respectively), indicating a CaMKII independent effect of O-GlcNAcylation on SOICR. Although the effect of Thm-G on SOICR was retained after CaMKII inhibition, +KN-93 abrogated the effect, increasing both F_SOICR_ and fractional release compared to cells treated with Thm-G without CaMKII inhibition (p = 0.0019 and p = 0.0084 for F_SOICR_ and fractional release, respectively). This indicates an additive effect of CaMKII and non-CaMKII O-GlcNAc mediated effects on SOICR. Interestingly, although it does not appear to translate to a change in the occurrence of SOICR (Fig. 3E), inhibition of CaMKII was still able to increase F_SOICR_ and fractional release in presence of Thm-G (p = 0.0019 and 0.0084 for F_SOICR_ and fractional release, respectively), suggesting CaMKII might still have a partial role under these conditions.

### The functional effect of O-GlcNAc of RyR2 is dependent on S2808

**Fig. 5:**
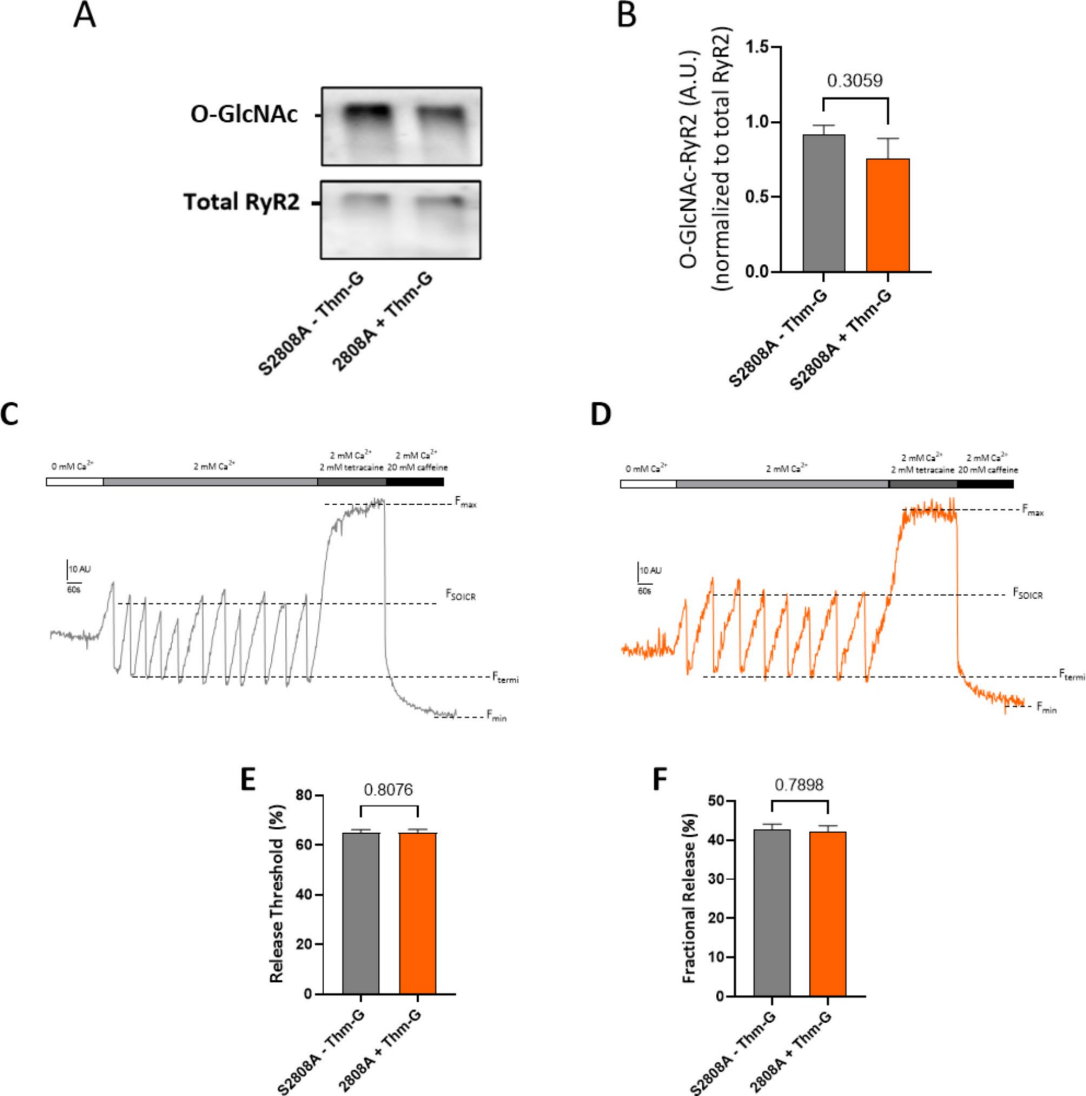
S2808 is required for CaMKII independent O-GlcNAcylation mediated changes in SOICR. (**A**) Representative blots showing O-GlcNAc-RyR2 and total RyR2. (**B**) Densitometric analysis of O-GlcNAc-RyR2 level. All O-GlcNAc-RyR2 was normalised to total RyR2. Results are shown as Mean ± SEM. n = 4 independent lysates. Statistical analysis was performed using an unpaired t-test. (**C**) Representative CEPIA traces for S2808 cells without Thm-G (**A**) and with Thm-G treatment (**D**). Comparison of (**E**) release threshold, and (**F**) fractional release in S2808 cells treated with or without Thm-G. Results were obtained from 125 and 135 cells (for – and + Thm-G, respectively) recorded over 8 independent experiments. To remove the impact of CaMKII, KN-93 was present in all conditions. Results are expressed as Mean ± SEM. Statistical analysis was performed using a Mann-Whitney test

Phosphorylation of RyR2 by PKA at S2808 has been shown to modify the function of RyR2. As phosphorylation and O-GlcNAcylation can modify the same amino acids and mediate similar effects in other proteins [10], we next explored whether O-GlcNAc modification of S2808 was responsible for the functional effect observed. We first examined the effect of preventing the O-GlcNAcylation at S2808. This was achieved using cells expressing a variant of RyR2 with serine at 2808 converted to alanine (S2808A) to prevent any O-linked modification at this site. As shown in Fig. 5A and B, conditions previously shown to increase RyR2 O-GlcNAcylation (25 mM glucose + Thm-G) were unable to increase RyR2 O-GlcNAcylation when S2808 was unavailable (p = 0.306). Next, we examined whether this prevention of O-GlcNAcylation was also able to prevent the O-GlcNAc mediated changes in SOICR. The representative traces are shown in Fig. 5 C and 5D while Fig. 5E F depict the release threshold and fractional release, respectively. Unlike in cells expressing wild-type RyR2, Thm-G + KN-93 had no effect on either F_SOICR_ (p = 0.807) or fractional release (p = 0.790) in cells expressing S2808A, indicating that the CaMKII independent effect of O-GlcNAcylation requires this site.

## Discussion

O-GlcNAcylation induced by hyperglycemia in diabetes mellitus is well established to have detrimental effects on Ca^2+^ handling in cardiomyocytes which can lead to arrhythmias [9, 12, 19]. Therefore, this study primarily investigated a direct role of O-GlcNAcylation of RyR2, a Ca^2+^ release channel frequently linked to arrhythmias is other settings. The significant findings of our study are: (1) RyR2 is post-translationally modified by O-GlcNAcylation; (2) hyperglycemia induced O-GlcNAcylation can increase SOICR through a CaMKII independent pathway via a reduction in the threshold for SOICR; and (3) S2808 is required for O-GlcNAcylation mediated changes in SOICR.

### RyR2 is modified by O-GlcNAcylation

O-GlcNAcylation is a ubiquitous post-translational modification, with an enormous number of proteins being identified as O-GlcNAcylated [20]. O-GlcNAcylation is well linked to cardiac disfunction [9]. RyR2 has previously been identified as a substrate of O-GlcNAcylation in the brain [14]; however, to the best of our knowledge it has never been assessed in the heart, the organ where RyR2 function is most well studied. Therefore, we first investigated whether cardiac expressed RyR2 is modified by O-GlcNAcylation. Our data show that RyR2 is O-GlcNAcylated in human RAA and rat left ventricles. Interestingly, although a clear trend is seen in both species, no significant difference in modification was observed in diabetes. Although unexpected, given hyperglycemia is a common driver of O-GlcNAcylation, this finding is consistent with prior observations that cardiac protein O-GlcNAcylation can be differentially regulated during diabetes [21]. Detecting a change in O-GlcNAcyation might have been hampered due to the large size of RyR2. A previous study of neuronally expressed RyR2 described a single O-GlcNAcylation site; threonine 1468 [14]. However, given the very large number of potential sites (575 within human RyR2, predicted by YinOYang 1.2 [22]) it seems unlikely this is the only one.

As other proteins commonly show differential changes at different sites occurring simultaneously, the overall integrated change does not reflect changes of individual sites [21, 23]. Additionally, in human RAA samples, the lack of discernible difference could also be due their relatively good glycemic control, with the diabetic patients in this study having a mean HbA1c of 56.9. Although we did not see a change in O-GlcNAcylation of RyR2 in diabetes, we were able to manipulate O-GlcNAcylation levels of RyR2 in HEK 293 cells. O-GlcNAcylation was increased under hyperglycemic conditions (25 mM glucose) and could be further increased using the promotor Thm-G. This suggests that RyR2 is O-GlcNAcylated under basal conditions but that under favorable conditions RyR2 can become overtly modified by O-GlcNAcylation.

### O-GlcNAcylation increases the propensity of SOICR by reducing the threshold for release

To determine the functional effect of O-GlcNAcylation of RyR2, we examined the occurrence of SOICR under the conditions we showed to modifiy the level of RyR2 O-GlcNAcylation. Under normoglycemic conditions promotion of O-GlcNAcylation by Thm-G had little effect on the occurrence of SOICR, presumably due to a limited amount of UDP-GlcNAc. However, a clear reduction in SOICR was observed when O-GlcNAcylation was inhibited. Under hyperglycemic conditions Thm-G caused a robust increase in the occurrence of SOICR at high extracellular Ca^2+^ concentrations. This effect on SOICR at higher Ca^2+^ levels is consistent with other promotors of SOICR such as phosphorylation and pro-SOICR drugs [5, 24]. At 25 mM glucose, the effect of O-GlcNAc inhibition was more pronounced with a dramatic reduction in SOICR occurrence. This is consistent with the corresponding drop in the level of O-GlcNAcylation. These changes in the occurrence of SOICR and correlation to changes in the O-GlcNAcylation of RyR2 suggest a relationship between the two. The ability of DON to reduce O-GlcNAcylation at both glucose concentrations indicates a basal level of RyR2 O-GlcNAcylation under normal conditions.

### O-GlcNAcylation can increase SOICR independent of CaMKII

The data above are consistent with our work in cardiac myocytes where we have previously shown O-GlcNAcylation promotes SOICR via CaMKII [12]. To dissect the CaMKII-independent effect, we repeated the SOICR experiments in the presence of KN-93. At lower extracellular Ca^2+^ concentrations KN-93 markedly reduced the occurrence of SOICR illustrating the previously observed influence of CaMKII on SOICR. However, at higher extracellular Ca^2+^ concentrations, CaMKII inhibition had no effect on the occurrence of SOICR indicating a CaMKII independent effect of O-GlcNAcylation. These data are interesting as they suggest that under conditions where SOICR is less common, O-GlcNAcylation is likely to induce SOICR via activation of CaMKII. However, under conditions where SOICR is already common, its occurrence can be further increased by O-GlcNAcylation independently of CaMKII. In many disease settings, such as diabetes, SOICR is already common due to excessive post-translational modifications such as phosphorylation and oxidation [4–7]. Therefore, it is likely that a CaMKII independent O-GlcNAcylation pathway becomes more relevant in a chronic disease state. As it appears that CaMKII and O-GlcNAcylation can regulate SOICR independently, these findings suggest inhibition of both pathways might be beneficial to prevent SOICR in metabolic disorders such as diabetes.

### CaMKII independent O-GlcNAc regulation of SOICR occurs due to a reduction in the release threshold

Our previous work has shown that a common mechanism leading to SOICR is a reduction in the threshold for Ca^2+^ release due to a sensitization of RyR2 to activation by luminal Ca^2+^. Similarly, the sensitivity of RyR2 to luminal Ca^2+^ also impacts when SOICR terminates and consequently, the fractional release [3, 15]. Using the luminally entrapped Ca^2+^ indicator CEPIA we found that CaMKII independent O-GlcNAc mediated regulation of SOICR (cells treated with KN-93 and Thm-G) also occurred due to a reduction in the release threshold. Interestingly, there was no change in the termination threshold, resulting in more but smaller SOICR events. This differential effect on release and termination thresholds is also shared by regulation of SOICR by FK506 binding proteins (FKBP12 and 12.6) which modify the termination threshold without effecting the release threshold and reduce the magnitude of SOICR [15]. Despite observing no significant effect of CaMKII activity on the occurrence of SOICR at high Ca^2+^ (1 mM) and high O-GlcNAc conditions, CaMKII was still able to modify F_SOICR_ and fractional release under similar conditions. The reason for the disparity is unclear but could be related to the already high incidence of SOICR and an insufficiently large increase in F_SOICR_ to impact when SOICR occurs. Nevertheless, our cytosolic and luminal Ca^2+^ measurements clearly demonstrate a CaMKII independent, O-GlcNAc mediated, increase in SOICR and corresponding decrease in F_SOICR_ and fractional release.

#### The functional effect of O-GlcNAcylation of RyR2 requires S2808

It is not unusual for O-GlcNAcylation to modify proteins at sites also phosphorylated [10]. Arguably the most well-studied phosphorylation site within RyR2 is S2808, a target of both PKA and CaMKII [8]. Therefore, we examined whether this site was also functionally relevant for regulation of RyR2 by O-GlcNAcylation. Surprisingly, given the large number of potential O-GlcNAcylation sites within RyR2, the removal of S2808 prevented the Thm-G mediated increase in RyR2 O-GlcNAcylation and abolished the CaMKII independent O-GlcNAcylation mediated regulation of RyR2. These findings indicate that S2808 represents the major O-GlcNAcylation sensitive functional site within RyR2. Although these data strongly implicate a direct modification of S2808 by O-GlcNAcylation, it does not rule out further indirect regulation of RyR2 activity by changes in kinases other than CaMKII. For example, we and others have shown that S2808 is a target of PKA and PKG [5, 8], so it remains possible that the loss of effect of O-GlcNAcylation on the S2808A mutant might be via changes in the activity of one of these kinases. However, in our hands, phosphorylation of S2808 has a modest impact on SOICR compared to the changes mediated by O-GlcNAcylation seen here [25]. This indicates that O-GlcNAcylation and not phosphorylation of this site might be the more functionally relevant modification. It also indicates that the balance between phosphorylation and O-GlcNAcylation might be important in the overall activity of RyR2. How O-GlcNAcylation of S2808 has a more overt effect on SOICR compared with phosphorylation is unclear. The region containing S2808 is poorly resolved in current structures, however, the bulkier sugar modification might disrupt the local structure more significantly that the smaller charged phosphate group. Future studies looking at these changes will be required, along with direct measurement of O-GlcNAcylation at S2808.

## Conclusion

In conclusion, our data are the first to demonstrate that O-GlcNAcylation can regulate RyR2 and SOICR independently of CaMKII. We also show this CaMKII independent regulation requires the S2808 residue, thus providing new knowledge to develop combinatorial strategies, via inhibition of both CaMKII and O-GlcNAcylation, to reduce SOICR and arrhythmias in diabetes. Specifically, the more prevalent role of CaMKII in inducing SOICR at lower Ca^2+^ levels suggests CaMKII inhibition might be the best strategy early in the disease (when SOICR is less likely), but in the later stages of diabetes when SOICR is more likely due to other triggers, such as oxidation, inhibition of O-GlcNAcylation might be more beneficial.

### Electronic supplementary material

Below is the link to the electronic supplementary material.

Supplementary Material 1

## Data Availability

The datasets used and/or analysed during the current study are available from the corresponding author on reasonable request.

## Abbreviations

RyR2: Ryanodine receptor II
CaMKII: Ca^2+^/calmodulin-dependent kinase II
HEK293: Human embryonic kidney 293
O-GlcNAc: O-GlcNAcylation
SOICR: Store overload-induced calcium release
Thm-G: Thiamet-G
DON: Diazo-6-oxornoleucine
CEPIA: Calcium-measuring organelle-entrapped protein indicators
