# Supplementary material for: Direct regulation of the cardiac ryanodine receptor (RyR2) by O-GlcNAcylation
Source: Cardiovasc Diabetol. 2023 Oct 13;22:276. doi: 10.1186/s12933-023-02010-3 (PMC10576323; doi:10.1186/s12933-023-02010-3)
Supplement: Supplementary file 1 — Supplementary Material 1 [file 12933_2023_2010_MOESM1_ESM.docx]

**Supplementary material**


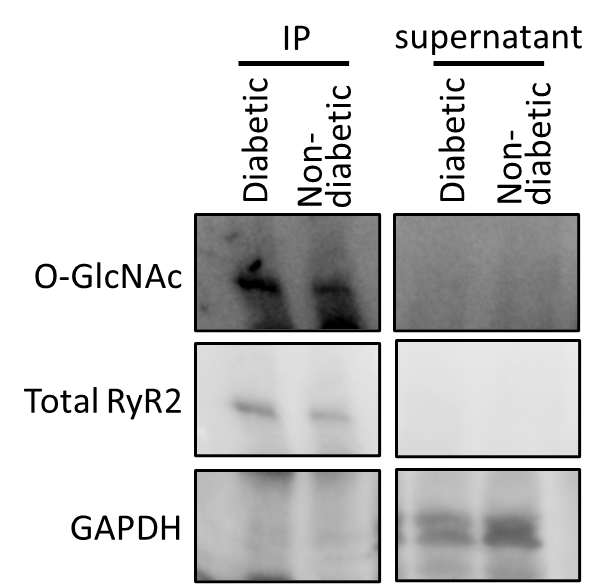


**Supplementary Figure S1.** Representative blot showing that RyR2 in diabetic and non-diabetic human RAA is modified by O-GlcNAcylation. RyR2 was immunoprecipitated (IP) from the samples using a total-RyR2 antibody and then probed using a total-RyR2 and total-O-GlcNAc antibodies. Supernatant is that removed from the corresponding IP samples.


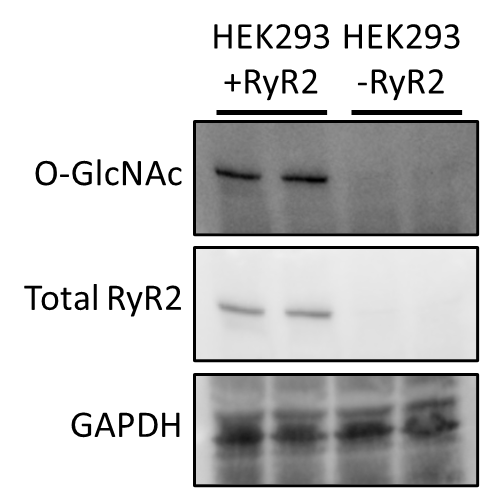


**Supplementary Figure S2.** Representative blot showing that RyR2 is modified by O-GlcNAcylation in HEK293 cells. Samples are HEK293 cells induced (+RyR2) or not induced (-RyR2) to express RyR2, probed with total-RyR2 and total-O-GlcNAc antibodies.
